# Prognosis of residual axillary disease after neoadjuvant chemotherapy in clinically node-positive breast cancer patients: isolated tumor cells and micrometastases carry a better prognosis than macrometastases

**DOI:** 10.1007/s10549-017-4157-0

**Published:** 2017-02-17

**Authors:** T. J. A. van Nijnatten, J. M. Simons, M. Moossdorff, L. de Munck, M. B. I. Lobbes, C. C. van der Pol, L. B. Koppert, E. J. T. Luiten, M. L. Smidt

**Affiliations:** 1 Department of Radiology and Nuclear Medicine, Maastricht University Medical Center+, P.O. Box 5800, 6202 AZ Maastricht, The Netherlands; 2 Department of Surgery, Maastricht University Medical Center+, Maastricht, The Netherlands; 3 GROW – School for Oncology and Developmental Biology, Maastricht University Medical Center+, Maastricht, The Netherlands; 4 Department of Surgery, University Medical Center Utrecht, Utrecht, The Netherlands; 5 Department of Research, Netherlands Comprehensive Cancer Organisation, Utrecht, The Netherlands; 6 Department of Surgical Oncology, Erasmus MC Cancer Institute, Rotterdam, The Netherlands; 7 Department of Surgery, Amphia Hospital, Breda, The Netherlands

**Keywords:** Breast cancer, Axillary lymph node, Neoadjuvant chemotherapy, Prognosis

## Abstract

**Purpose:**

The aim of this study was to compare disease-free survival (DFS) and overall survival (OS) between clinically node-positive breast cancer patients, treated with neoadjuvant chemotherapy (NAC), with axillary pathologic complete response (ypN0), residual axillary isolated tumor cells or micrometastases (ypNitc/mi), and residual axillary macrometastases (ypN1-3).

**Methods:**

All patients diagnosed with clinically node-positive primary invasive breast cancer treated with NAC and subsequent axillary lymph node dissection between 2005 and 2008 were retrospectively analyzed. Data were obtained from the Netherlands Cancer Registry. Patients were stratified by final pathological axillary status: ypN0, ypNitc/mi, or ypN1-3. The main outcome measures DFS and OS were analyzed using Kaplan–Meier survival analysis. Uni- and multivariable cox regression analyses were used to determine independent predictors for DFS and OS.

**Results:**

A total of 1347 patients were included. Pathologic nodal status was ypN0 in 22.2%, ypNitc/mi in 3.8%, and ypN1-3 in 74.0% of patients. Overall, 5-year DFS was 57.8% and mean OS was 7.4 years. DFS and OS were comparable between ypN0 and ypNitc/mi (HR 1.38 (0.40–4.79, *p* = 0.613) and HR 0.92 (0.27–3.09, *p* = 0.889), respectively), but significantly different between ypN0 and ypN1-3 (HR 1.78 (1.06–3.00, *p* = 0.031) and HR 1.70 (1.07–2.71, *p* = 0.026), respectively).

**Conclusions:**

Clinically node-positive patients, treated with NAC, with axillary nodal status ypN0 or ypNitc/mi carry similar prognosis regarding DFS and OS. Axillary nodal status ypN1-3 is associated with a less favorable prognosis. Future studies should consider ypN0 and ypNitc/mi as one entity.

## Introduction

Over the past 20 years, a trend toward a less invasive approach regarding the surgical management of the axilla in breast cancer patients has been observed. Nowadays, a sentinel lymph node biopsy (SLNB) has been widely adopted for staging of early-stage clinically node-negative breast cancer [1]. In case of a sentinel lymph node (SLN) containing isolated tumor cells (ITCs) or micrometastases, a completion of axillary lymph node dissection (ALND) does not improve survival, nor does it reduce regional recurrence. Consequently, ALND following SLNB has been abandoned in these patients [2–4]. The ACOSOG Z0011 trial demonstrated no significant effect on prognosis when ALND is omitted in case of a SLN containing a limited number of metastases, even macrometastases, in patients treated with breast conserving therapy [3].

In clinically node-positive (cN+) patients, ALND is regarded as standard surgical therapy. However, increased utilization of neoadjuvant chemotherapy (NAC) results in axillary pathologic complete response (pCR) in 30–40% of patients [5]. Consequently, the value of ALND is topic of debate. Various studies demonstrated that axillary pCR after NAC is associated with improved prognosis [6–8]. Residual axillary disease has a less favorable prognosis, but it is unknown whether different degrees of residual axillary disease (i.e., ITCs, micrometastases, macrometastases) all have similar prognosis.

Hence, the purpose of this study was to compare prognosis of axillary pCR, residual ITCs, or micrometastases and residual macrometastases in cN+ patients treated with NAC.

## Methods

### Data collection

In this study, all pathologically confirmed cN+ patients diagnosed with primary invasive breast cancer and treated with NAC (with or without immunotherapy) followed by ALND between 2005 and 2008 were included. Exclusion criteria were synchronous breast cancer, primary surgical treatment, neoadjuvant radiation therapy, neoadjuvant endocrine therapy, unknown pathological nodal status, and distant metastases diagnosed within 91 days after primary breast cancer diagnosis. Patients who did not undergo ALND were also excluded.

Data were obtained from the Netherlands Cancer Registry (NCR), which is managed by the Netherlands Comprehensive Cancer Organisation (NCCO). The PALGA foundation (Pathologisch-Anatomisch Landelijk Geautomatiseerd Archief), a nationwide network and registry of histopathology and cytopathology diagnosis in the Netherlands, regularly submits reports of all diagnosed malignancies to the cancer registry. After notification, trained data collection registrars from the NCR extracted data from patients’ records. Data were collected on age, tumor type, receptor status, surgical procedures, systemic therapy, adjuvant radiation therapy, and pathology results, including pathological TNM stage and tumor grade. During a 5-year period after initial diagnosis, the first of the following breast cancer events was registered: any local, regional, or contralateral recurrence or distant metastasis. Date of death or date of emigration was derived from the Municipal Personal Records Database (Basisregistratie Personen, BRP) and files until December 31, 2014 were analyzed.

Patients were stratified into three subgroups according to final pathologic axillary nodal status after completion of NAC and definitive surgery: pCR (ypN0), residual isolated tumor cells or micrometastases (ypNitc/mi), and residual macrometastases (ypN1-3).

### Neoadjuvant chemotherapy (NAC) with/without immunotherapy regimen

During the study period, the Dutch national guideline of 2005 was in use [9]. This guideline recommended chemotherapy regimens consisting of five courses 5 Fluorouracil, Epirubicin, Cyclophosphamide (FEC), or six courses of Taxotere, Adriamycin, and Cyclophosphamide (TAC). In case of Her2Neu receptor (Her2) amplification, targeted therapy (trastuzumab) was recommended in addition to chemotherapy.

### Statistics

Statistical analyses were performed using Statistical Package for the Social Sciences software (Version 22, IBM, Armonk, New York, USA). General characteristics between the three subgroups were compared using Chi squared test for categorical data and One-way ANOVA for continuous data, after confirmation of Levene’s test for equality of variances. If Levene’s test demonstrated significant differences among the population variances, Kruskall–Wallis test was used.

DFS was defined as time from diagnosis to any local (including carcinoma in situ), regional, or contralateral recurrence, distant metastasis or mortality within 5 years after the primary diagnosis. Events occurring 0–91 days after diagnosis were considered synchronous to the original tumor and were not counted as recurrence. OS was defined as the time interval between date of diagnosis and date of death, date of first event, date of last follow-up, or date of emigration.

DFS and OS for the three subgroups were calculated with Kaplan–Meier curves and compared with the log-rank test. *p* values (two-sided) <0.05 were considered statistically significant. Relevant clinicopathological variables associated with DFS and OS were examined using univariable and, where applicable, multivariable Cox proportional hazards regression, with Hazard Ratio (HR) and corresponding 95% confidence intervals.

## Results

**Fig. 1:**
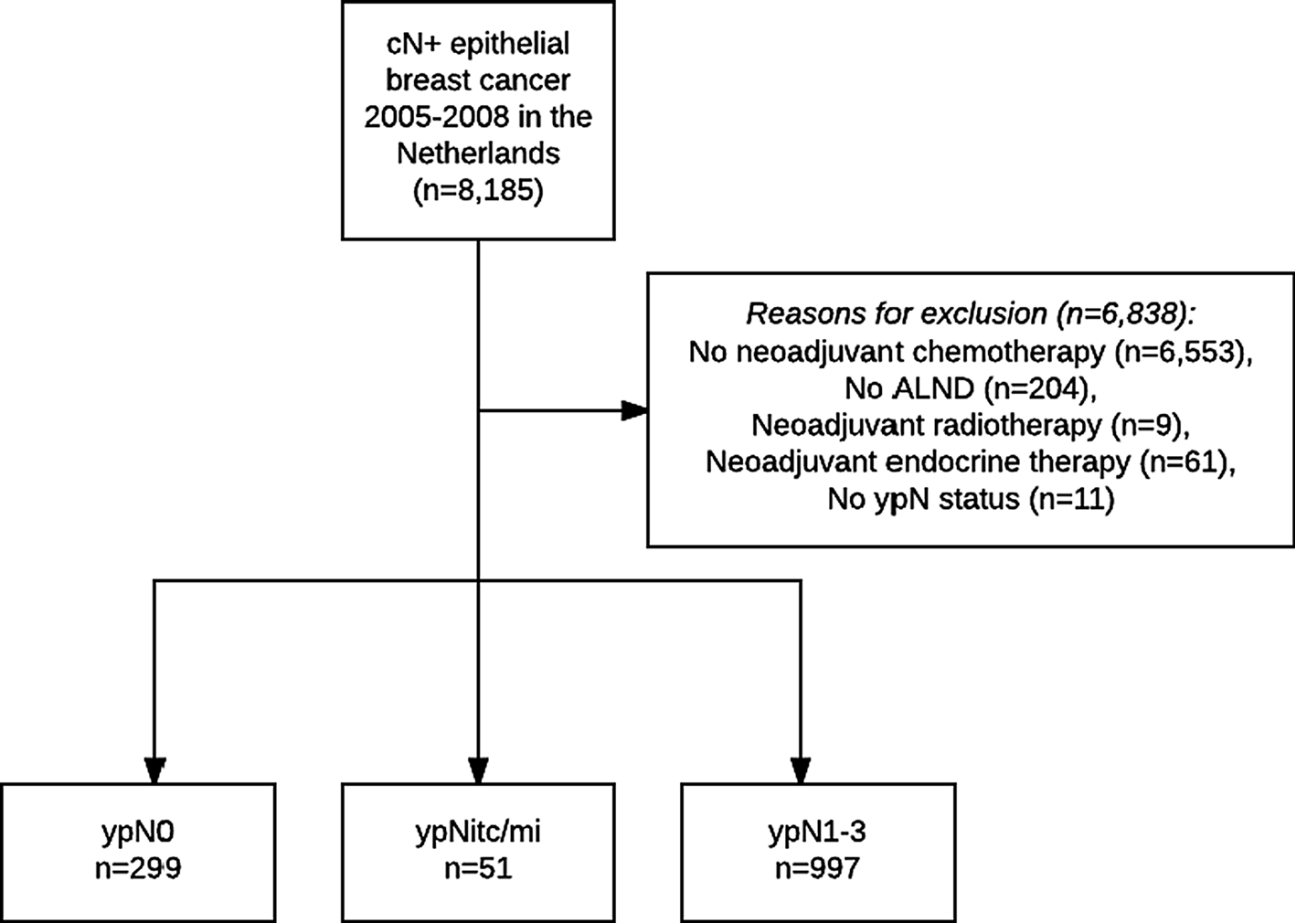
Flowchart of included patients. *cN*+ clinically node-positive status, *SLNB* sentinel lymph node biopsy, *ALND* axillary lymph node dissection, *ypN0* axillary pathologic complete response, *ypNitc/mi* axillary residual isolated tumor cells or micrometastases, *ypN1*-*3* axillary residual macrometastases

Between 2005 and 2008, 8176 patients were diagnosed with cN+ breast cancer in the Netherlands. Patients were excluded for several reasons: 6553 patients underwent primary surgery; 204 patients did not undergo ALND; 9 patients were treated with neoadjuvant radiotherapy; 61 patients were treated with neoadjuvant endocrine therapy; and ypN status was unknown for 11 patients (Fig. 1). A final total of 1347 patients were included for this study: 299 ypN0, 51 ypNitc/mi and 997 ypN1-3.

The incidence of pCR of the primary tumor was higher in patients with ypN0 compared to ypNitc/mi and ypN1-3 patients (41.1 vs. 19.6 and 7.1%, respectively, *p* < 0.001). Furthermore, lobular carcinoma was observed more often in patients with ypN1-3 than in ypN0 and ypNitc/mi patients (9.4 vs. 5.4 and 3.9%, respectively, *p* = 0.039). Adjuvant radiation therapy was applied more often in ypN1-3 as compared to ypN0 and ypNitc/mi patients (92.1 vs. 80.9 and 80.4%, respectively, *p* < 0.001, Table 1).

**Table 1:** General characteristics

	ypN0 (*n* = 299)	ypNitc/mi (*n* = 51)	ypN1-3 (*n* = 997)	*p* value
Mean age (years) (range)	48.9 (27–77)	48.2 (29–81)	50.4 (22–85)	0.053
Clinical T-stage (%)				
cT0-is	1 (0.3)	0	5 (0.5)	0.826
cT1-2	138 (47.0)	26 (52.0)	458 (47.0)	0.780
cT3-4	153 (52.0)	24 (48.0)	509 (52.0)	0.853
cTx	7	1	25	–
Pathologic T-stage (%)				
ypT0-is	123 (50.8)	10 (26.3)	71 (8.5)	<0.001
ypT1-2	107 (44.2)	25 (65.8)	570 (68.5)	<0.001
ypT3-4	12 (5.0)	3 (7.9)	191 (23.0)	<0.001
Unknown	57	13	165	–
Tumor grade (%)				
1–2	28 (31.5)	7 (50.0)	197 (43.9)	<0.001
3	61 (68.5)	7 (50.0)	252 (56.1)	0.051
Unknown	210	37	548	–
Tumor type (%)				
Ductal	227 (75.9)	41 (80.4)	746 (74.8)	0.739
Lobular	16 (5.4)	2 (3.9)	94 (9.4)	0.039
Other^a^	56 (18.7)	8 (15.7)	157 (15.8)	0.470
Subtype (%)				
ER+PR+ , Her2−	35 (12.8)	11 (22.5)	349 (37.3)	<0.001
ER+PR−, Her2−	21 (7.7)	7 (14.3)	122 (13.0)	0.035
ER+Her2+	47 (17.1)	20 (40.8)	152 (16.3)	<0.001
ER−Her2+	97 (35.4)	5 (10.2)	146 (15.6)	<0.001
Triple negative	74 (27.0)	6 (12.2)	166 (17.8)	0.003
Unknown	25	2	62	–
Breast surgery (%)				
Breast conserving therapy	62 (20.7)	12 (23.5)	181 (18.2)	0.421
Mastectomy	237 (79.3)	39 (76.5)	816 (81.8)	0.421
Unknown	0	0	1	–
Radiation therapy (%)				
Yes	242 (80.9)	41 (80.4)	918 (92.1)	<0.001
Endocrine therapy to ER+ subtype (%)				
Yes	95 (84.8)	35 (92.1)	600 (91.5)	0.080
Trastuzumab to Her2+ subtype (%)				
Yes	127 (92.0)	19 (76.0)	247 (89.2)	0.057

*ypN0* axillary pathologic complete response, *ypNitc/mi* axillary residual isolated tumor cells or micrometastases, *ypN1*-*3* axillary residual macrometastases, *cT*-*stage* clinical tumor stage, *pT*-*stage* pathologic tumor stage, *ER* estrogen, *PR* progesterone, *Her2* human epidermal growth factor receptor 2

^a^Including adenocarcinoma not otherwise specified, mucinous carcinoma, and mixed carcinoma

### Disease-free survival

**Fig. 2:**
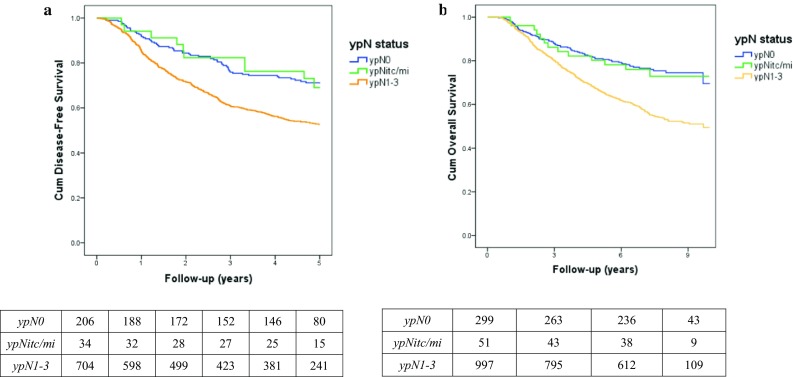
Kaplan–Meier curves for disease-free (**a**) and overall survival (**b**), including number at risk. *ypN status* pathologic nodal status after neoadjuvant chemotherapy, *ypN0* axillary pathologic complete response, *ypNitc/mi* axillary residual isolated tumor cells or micrometastases, *ypN1*-*3* axillary residual macrometastases

Five-year follow-up was available for 944 patients (70.1%; *n* = 206 ypN0, *n* = 34 ypN0i+/ypN1mi, *n* = 704 ypN1-3): Recurrence occurred in 377 patients (39.9%) and 22 patients died within 5 years (2.3%). This resulted in a DFS event in 42.2% of the patients. DFS did not differ significantly between ypN0 and ypNitc/mi (71.8 vs. 70.6%, *p* = 0.978). When DFS was compared between ypN0 and ypN1-3, a significant difference was found (71.8 vs. 53.4%; *p* = 0.049) (Fig. 2a).

Multivariable Cox regression analyses demonstrated no significant difference in DFS between ypN0 and ypNitc/mi (HR 1.38 (0.40–4.79), *p* = 0.613), but a significant difference in DFS between ypN0 and ypN1-3 (HR 1.78 (1.06–3.00), *p* = 0.031) (Table 2).

**Table 2:** Uni- and multivariable analyses of predictors of disease-free survival at 5 years

	Univariable analysis		Multivariable analysis	
	HR (95% CI)	*p* value	HR (95% CI)	*p* value
ypN0	Reference	0.964	Reference	0.613
ypNitc/mi	1.02 (0.52–1.99)	<0.001	1.38 (0.40–4.79)	0.031
ypN1-3	1.89 (1.43–2.50)		1.78 (1.06–3.00)	
Age (per year increment)	1.02 (1.01–1.02)	0.001	1.01 (0.99–1.02)	0.479
ypT-stage				
T0 or Tis	Reference		Reference	
T1-2	1.88 (1.31–2.70)	0.001	2.73 (1.39–5.39)	0.004
T3-4	3.74 (2.53–5.54)	<0.001	4.71 (2.35–9.43)	<0.001
Tumor type				
Ductal	Reference		Reference	
Lobular	0.78 (0.53–1.14)	0.193	1.11 (0.59–2.07)	0.751
Other	0.89 (0.69–1.15)	0.386	0.89 (0.58–1.37)	0.595
Tumor grade				
3 versus 1–2	1.64 (1.22–2.20)	0.001	1.69 (1.19–2.40)	0.004
Subtypes				
ER+PR+Her2−: yes versus no^a^	0.63 (0.50–0.80)	<0.001		
ER+PR−Her2−: yes versus no^a^	1.32 (0.99–1.75)	0.057		
ER+Her2+: yes versus no^a,b^	0.63 (0.47–0.85)	0.002		
ER−Her2+: yes versus no^b^	1.20 (0.95–1.53)	0.129		
Triple negative: yes versus no	1.94 (1.46–2.32)	<0.001	1.16 (0.69–1.93)	0.577
Trastuzumab				
Yes versus no	0.81 (0.65–1.00)	0.052	0.76 (0.47–1.23)	0.263
Endocrine therapy				
Yes versus no	0.57 (0.47–0.69)	<0.001	0.55 (0.36–0.85)	0.007
Radiation therapy				
Yes versus no	1.09 (0.78–1.52)	0.626	0.74 (0.45–1.23)	0.251

*HR* hazard ratio, *ypN0* axillary pathologic complete response, *ypNitc/mi* axillary residual isolated tumor cells or micrometastases, *ypN1*-*3* axillary residual macrometastases, y*pT*-*stage* pathologic tumor stage after neo-adjuvant chemotherapy, *ER* estrogen, *PR* progesterone, *Her2* human epidermal growth factor receptor 2

^a^Excluded from multivariable analysis due to collinearity with endocrine therapy

^b^Excluded from multivariable analysis due to collinearity with trastuzumab

Furthermore, higher ypT stage (ypT 1-2: HR 2.73 (1.39–5.39), *p* = 0.004 and ypT 3-4: HR 4.71 (2.35–9.43), *p* < 0.001) and higher tumor grade (HR 1.69 (1.19–2.40), *p* = 0.004) were identified as independent predictors of decreased DFS, whereas endocrine therapy was identified as independent predictor of increased DFS (HR 0.55 (0.36–0.85), *p* = 0.007).

### Overall survival

Mean OS was 7.4 years (range 0.4–10 years): 8.3 years for ypN0, 8.2 years for ypNitc/mi, and 7.0 years for ypN1-3 (Fig. 2b). OS was comparable between ypN0 and ypNitc/mi (*p* = 0.875). However, OS was significantly lower for ypN1-3 as compared to ypN0 (*p* = 0.014).

Multivariable Cox regression analyses demonstrated no significant difference in OS between ypN0 and ypNitc/mi (HR: 0.92 (0.27–3.09), *p* = 0.889), but a significant difference in OS between ypN0 and ypN1-3 (HR 1.70 (1.07–2.71)), *p* = 0.026) (Table 3).

**Table 3:** Uni- and multivariable analyses of predictors for overall survival

	Univariable analysis		Multivariable analysis	
	HR (95% CI)	*p* value	HR (95% CI)	*p* value
ypN0	Reference	0.816	Reference	0.889
ypNitc/mi	1.07 (0.59–1.94)	<0.001	0.92 (0.27–3.09)	0.026
ypN1-3	2.07 (1.62–2.67)		1.70 (1.07–2.71)	
Age (per year increment)	1.02 (1.02–1.03)	<0.001	1.01 (1.00–1.02)	0.082
ypT-stage				
T0 or Tis	Reference	0.002	Reference	
T1-2	1.60 (1.19–2.15)	<0.001	2.40 (1.32–4.36)	0.004
T3-4	3.33 (2.41–4.59)		4.38 (2.37–8.12)	<0.001
Tumor type				
Ductal	Reference		Reference	
Lobular	0.98 (0.72–1.33)	0.906	1.33 (0.78–2.27)	0.288
Other	0.98 (0.78–1.24)	0.882	1.03 (0.69–1.53)	0.887
Tumor grade				
3 versus 1–2	1.84 (1.41–2.40)	<0.001	1.72 (1.25–2.36)	0.001
Subtypes				
ER+PR+Her2−: yes versus no^a^	0.62 (0.51–0.76)	<0.001		
ER+PR−Her2−: yes versus no^a^	1.28 (1.00–1.65)	0.050		
ER+Her2+: yes versus no^a,b^	0.58 (0.43–0.73)	<0.001		
ER−Her2+: yes versus no^b^	1.15 (0.93–1.42)	0.201		
Triple negative: yes versus no	2.10 (1.73–2.56)	<0.001	1.38 (0.90–2.13)	0.145
Trastuzumab				
Yes versus no	0.72 (0.59–0.88)	0.001	0.70 (0.46–1.07)	0.101
Endocrine therapy				
Yes versus no	0.56 (0.47–0.66)	<0.001	0.49 (0.34–0.72)	<0.001
Radiation therapy				
Yes versus no	1.31 (0.96–1.77)	0.086	1.06 (0.64–1.74)	0.829

*HR* hazard Ratio, *ypN0* axillary pathologic complete response, *ypNitc/mi* axillary residual isolated tumor cells or micrometastases, *ypN1*-*3* axillary residual macrometastases, y*pT*-*stage* pathologic tumor stage after neo-adjuvant chemotherapy, *ER* estrogen, *PR* progesterone, *Her2* human epidermal growth factor receptor 2

^a^Excluded from multivariable analysis due to collinearity with endocrine therapy

^b^Excluded from multivariable analysis due to collinearity with trastuzumab

Other independent predictors of decreased OS were higher ypT stage (ypT 1-2: HR 2.40 (1.32–4.36), *p* = 0.004) and ypT 3-4: HR 4.38 (2.37–8.12), *p* < 0.001) and higher tumor grade (HR 1.72 (1.25–2.36), *p* = 0.001). Furthermore, endocrine therapy (HR 0.49 (0.34–0.72), *p* < 0.001) was identified as an independent predictor of increased OS.

## Discussion

This is the first study comparing prognosis of ypN0 with ypNitc/mi and ypN1-3 in cN+ breast cancer patients treated with NAC. It is well known that axillary pCR is an important prognostic factor [6–8]. Residual axillary disease after completion of NAC is associated with a less favorable prognosis. However, to our knowledge, this is the first study that compares the long-term effect of different degrees of residual disease on prognosis. Our study showed that ypN0 and ypNitc/mi carry similar prognosis and that ypN1-3 carries a significantly different and less favorable prognosis in terms of DFS and OS.

Current guidelines still recommend to perform ALND in cN+ patients following NAC irrespective of axillary response [10, 11]. However, cN+ patients converting to axillary pCR after completion of NAC remain a topic of debate since they are not expected to benefit from ALND. A non-invasive technique to accurately diagnose pCR is currently unavailable. Various minimally invasive procedures have been suggested for this purpose. The SLNB was studied extensively and its reliability seems questionable with a reported overall false negative rate (FNR) of 15.1% and negative predictive values (NPV) of 86% or lower [5]. Other recently introduced minimally invasive techniques, the MARI procedure (Marking the Axillary lymph node with Radioactive Iodine seeds) and TAD (Targeted Axillary Dissection), are promising with FNRs of 7 and 2%, respectively. However, with only evidence available of single center studies comprising small cohorts that support these techniques it is not (yet) safe to implement them in clinical practice [12, 13].

In our cohort, all patients underwent an ALND and thus our results do not directly support a change in surgical axillary treatment after the completion of NAC. Considering the comparable prognosis between ypN0 and ypNitc/mi, our results do question whether ypNitc/mi may mimic ypN0 more than residual axillary disease. Thus, when minimally invasive procedures prove to predict the status of the axilla accurately, the indications for omitting ALND may not just be limited to ypN0. Therefore, current research on reducing axillary management in cN+ patients should not focus only on ypN0 patients, but also on patients with ypNitc/mi. In future, ALND may be rendered as a procedure only to manage residual macrometastases.

In clinically node-negative patients in adjuvant setting, the SLNB with a relatively high FNR of about 8% is permitted since axillary recurrences are rare and previous studies have shown that not all axillary residual disease eventually converts to clinically overt disease [2, 3, 14]. This is in part effectuated by adjuvant therapy (i.e., radiation and/or systemic therapy) and by biological subtypes influencing recurrence patterns. In cN+ patients, however, no studies have adequately evaluated prognostic impact of omitting ALND in case of residual axillary disease. Despite this, a trend toward replacing ALND by less invasive axillary staging procedures that are known to miss potentially therapy-resistant disease is already ongoing worldwide. Therefore, it is of utmost importance to prospectively collect data of these patients to detect potential influences on prognosis.

Since prognosis seems comparable between post-ALND ypN0 and ypNitc/mi in cN+ patients treated with NAC, imaging might play an important role in axillary staging after NAC in the future. Since ITCs and micrometastases are not detectable on high-resolution exams, such as MRI or 18F-FDG PET/CT, imaging techniques were considered inaccurate for nodal assessment after completion of NAC. Yet, with our current observations in mind, dedicated axillary imaging is re-entering the arena as a modality to non-invasively identify residual macrometastases rather than ‘any’ extent of residual disease (including ITCs and micrometastases).

The strength of the current study is the large cohort of patients that all underwent ALND after NAC. But our study also has several limitations. Subgroups ypN0 and ypN1-3 comprised 299 and 997 patients, respectively, where subgroup ypNitc/mi comprised only 51 patients. Our ypNitc/mi subcohort was too small to explore the influence of single versus multiple tumor-positive lymph nodes on prognosis, and further studies are needed to explore this concept. Yet, this subset of patients can be considered unique since ypNitc/mi in cN+ breast cancer is rare and a previously reported study included only a few ypNitc/mi patients [15].

Furthermore, our cohort was treated up to a decade ago. In that time frame, different guidelines were effective, and therefore results should be interpreted carefully regarding current practice. For example, some Her2+ patients did not receive trastuzumab in our cohort (19.6%), since trastuzumab was just introduced by that time.

Finally, our results are based on a retrospective study design. Consequently, details on additional radiation therapy could not be taken into account since radiation therapy fields were not recorded for each patient. Therefore, its influence on prognosis could not be explored in more detail.

In conclusion, our study showed that prognosis of cN+ patients who receive NAC is affected by the degree of axillary residual disease as measured in ALND specimens. Prognosis of isolated tumor cells and micrometastases was comparable to prognosis of ypN0 and more favorable than prognosis of macrometastases in terms of DFS and OS irrespective of tumor type. Ongoing and future studies should therefore consider ypN0 and ypNitc/mi as one entity. Future research must explore which patients may safely receive a different, less invasive approach than the current standard of performing ALND after completion of NAC in all patients that were cN+ prior to NAC.
